# Galectin-3 in Peripheral Artery Disease. Relationships with Markers of Oxidative Stress and Inflammation

**DOI:** 10.3390/ijms18050973

**Published:** 2017-05-04

**Authors:** Isabel Fort-Gallifa, Anna Hernández-Aguilera, Anabel García-Heredia, Noemí Cabré, Fedra Luciano-Mateo, Josep M. Simó, Vicente Martín-Paredero, Jordi Camps, Jorge Joven

**Affiliations:** 1 Biochemical Research Unit, Hospital Universitari de Sant Joan, Institut d’Investigació Sanitària Pere Virgili, Universitat Rovira i Virgili, C. Sant Joan s/n, Reus, 43201 Catalonia, Spain; ifort@lrsud.cat (I.F.-G.); anna.hernandeza@gmail.com (A.H.-A.); ghanabel@gmail.com (A.G.-H.); noemi.cabre@gmail.com (N.C.); fedra.luciano@gmail.com (F.L.-M.); jjoven@grupsagessa.com (J.J.); 2 Reference Laboratory of Catalonia South, Hospital Universitari de Sant Joan, Institut d’Investigació Sanitària Pere Virgili, Universitat Rovira i Virgili, Av. Cambra de Comerç 42, Reus, 43204 Catalonia, Spain; jmsimo@lrsud.cat; 3 Service of Angiology, Vascular Surgery and Endosurgery, Hospital Universitari Joan XXIII, Institut d’Investigació Sanitària Pere Virgili, Universitat Rovira i Virgili, C. Dr. Mallafré Guasch 4, Tarragona, 43005 Catalonia, Spain; vparedero.hj23.ics@gencat.cat

**Keywords:** atherosclerosis, F2-isoprostanes, galectin-3, oxidative stress, peripheral artery disease

## Abstract

Galectin-3 is a modulator of oxidative stress, inflammation, and fibrogenesis involved in the pathogenesis of vascular diseases. The present study sought to characterize, in patients with peripheral artery disease (PAD), the localization of galectin-3 in arterial tissue, and to analyze the relationships between the circulating levels of galectin-3 and oxidative stress and inflammation. It also sought to compare the diagnostic accuracy of galectin-3 with that of other biochemical markers of this disease. We analyzed femoral or popliteal arteries from 50 PAD patients, and four control arteries. Plasma from 86 patients was compared with that from 72 control subjects. We observed differences in the expression of galectin-3 in normal arteries, and arteries from patients with PAD, with a displacement of the expression from the adventitia to the media, and the intima. In addition, plasma galectin-3 concentration was increased in PAD patients, and correlated with serologic markers of oxidative stress (F2-isoprostanes), and inflammation [chemokine (C−C motif) ligand 2, C-reactive protein, β-2-microglobulin]. We conclude that the determination of galectin-3 has good diagnostic accuracy in the assessment of PAD and compares well with other analytical parameters currently in use.

## 1. Introduction

Galectins are a lectin family able to bind to β-galactoside groups [1]. To-date, 14 mammalian galectins have been identified, all of which contain a carbohydrate-recognition-binding domain of 130 amino acids. Galectin-3 is a 29- to 35-kDa protein consisting of two domains, the C-terminal carbohydrate recognition binding domain, and the N-terminal domain that has a unique short end continuing into a Pro-Gly-Ala-Tyr-rich repeat motif [2]. Galectin-3 has been recognized increasingly as a modulator of multiple biological functions such as oxidative stress, proliferation, macrophage chemotaxis, phagocytosis, neutrophil extravasation, neutrophil migration during venous thrombosis, apoptosis, vacuole lysis after infection, fibrogenesis, and angiogenesis [3,4,5,6]. Relationships with oxidative stress have been demonstrated in vitro, such that treatment of monocytes with phorbol myristate acetate, a nicotinamide adenine dinucleotide phosphate (NADPH) oxidase-dependent inducer of reactive oxygen species produced an increase in galectin-3 mRNA and protein expression, while blocking with apocynin reversed these effects [7].

Recent evidence suggests that galectin-3 plays a role in the pathogenesis of numerous disease conditions including cancer, as well as inflammatory and metabolic disorders [8,9,10,11]. The role of galectin-3 in atherosclerosis deserves special attention. Inactivation of galectin-3 gene or therapeutic modulation of the protein levels was shown to halt the progression of cardiac remodeling, attenuate myocardial fibrogenesis, reduce the atherosclerotic lesion size, and preserve ventricular function in rats and mice [12,13,14,15]. Further, galectin-3 is involved in vascular smooth muscle cell osteogenic differentiation [16], myocardial fibrosis and inflammation [17], together with a strong suggestion of association with the risk of cardiac fibrosis, hearth failure, and mortality in the general population, and in patients with atherosclerosis [7,18,19,20].

In contrast to cardiac disease, information on galectin-3 in peripheral artery disease (PAD) is scarce. Lower-extremity PAD is a frequent manifestation of atherosclerosis that is associated with extensive impairment of different arterial territories. The prevalence of this disease increases with age and in people over the age of 70 years it is estimated to be about 20% [21]. Oxidative stress and inflammation are important factors for the initiation and progression of PAD, and the inflammatory mediators involved in this process are similar to those contributing to coronary artery disease [22,23]. A differential characteristic of PAD is that the associated atherosclerosis affects more extensive regions of arteries and, consequently, the associated biochemical alterations are often more marked than in coronary artery disease [24]. The possibility of finding efficient, non-invasive biomarkers for the early diagnosis of PAD is currently being investigated. Several studies proposed C-reactive protein (CRP) measured by a high-sensitivity method, or β-2-microglobulin (B2M), as useful markers of this disease [25,26]. We recently reported that the measurement of the serum concentrations of F2-isoprostanes and/or the chemokine (C−C motif) ligand 2 (CCL2), markers of oxidative stress and inflammation, may also constitute excellent biomarkers for the diagnosis of PAD [27].

The present study characterized the localization of galectin-3 in arterial tissue, and evaluated the relationships between the circulating levels of galectin-3 versus oxidative stress and inflammation. The diagnostic accuracy of galectin-3 compared favorably with other biochemical markers of these processes in patients with PAD, relative to control individuals.

## 2. Results

### 2.1. Histological and Immunohistochemical Analyses

**Figure 1:**
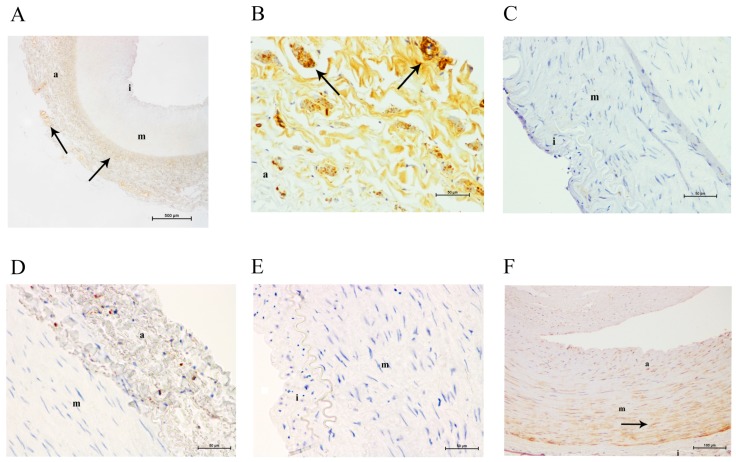
Immunohistochemical images of a normal femoral artery. (**A**) Galectin-3 staining in adventitia at a magnification of 40×; (**B**) galectin-3 staining in the adventitia at a magnification of 200×; (**C**) lack of galectin-3 staining in tunica media at a magnification of 200×; (**D**) faint CD68 staining in the adventitia at 200× magnification; (**E**) lack of CD68 staining in the media at 200× magnification; (**F**) α-actin staining in the media at 100× magnification. **a**, adventitia; **m**, media; **i**, intima. The arrows show positive immunostained areas.

In normal arteries, galectin-3 expression was evenly distributed throughout the adventitia of the artery wall. We observed increased staining in certain areas, coinciding with inflammatory infiltrates. We did not see any specific staining in the tunica media or the tunica intima. We observed a faint positive labeling for CD68 antigen (a marker of macrophages) in the adventitia, but not in the media or the intima, and a positive labeling for α-actin (a marker of smooth muscle cells) in the media (Figure 1).

**Figure 2:**
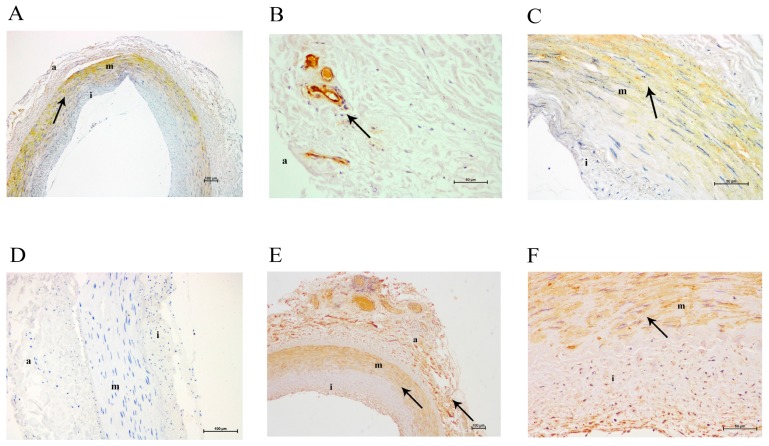
Immunohistochemical images of a moderately altered femoral artery. (**A**) Galectin-3 staining at 40× magnification; (**B**) galectin-3 staining in adventitia at 200× magnification; (**C**) galectin-3 staining in tunica media at 200× magnification; (**D**) lack of CD68 staining at 100× magnification; (**E**) α-actin staining at 40× magnification; (**F**) α-actin staining at 200× magnification. **a**, adventitia; **m**, media; **i**, intima. The arrows show positive immunostained areas.

**Figure 3:**
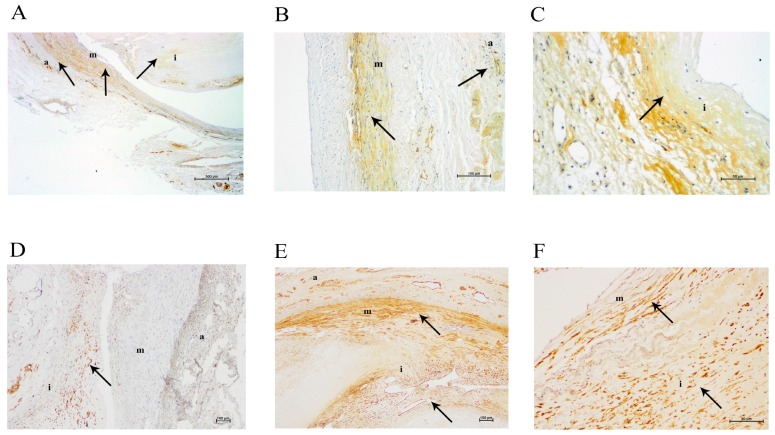
Immunohistochemical images of a severely altered femoral artery. (**A**) Galectin-3 staining at 40× magnification; (**B**) galectin-3 staining in adventitia at 100× magnification; (**C**) galectin-3 staining in tunica intima at 200× magnification; (**D**) CD68 staining at 100×magnification; (**E**) α-actin staining in adventitia, media, and intima at 100× magnification; (**F**) α-actin staining in media and intima at 200× magnification. **a**, adventitia; **m**, media; **i**, intima. The arrows show positive immunostained areas.

Arterial samples from patients with PAD had an increased intima thickness with respect to the media (intima/media ratio: 2.06 (0.52–6.19) versus 0.13 (0.10–0.16); *p* = 0.002). Subjects with the less thickened intima layer showed galectin-3 localization mainly together with smooth muscle cells of the media and some positive staining in the remains of inflammatory infiltrates around the vessels of the adventitia, but not in the intima (Figure 2)*.* In contrast, arteries from patients with the more thickened intima showed staining for galectin-3, CD68 and α-actin in the intima (Figure 3).

There were no significant differences in the percentage positive staining for galectin-3 between patients and controls [3.55% (0.60–13.69)% versus 7.11% (0.93–11.04)%, respectively; *p* = 0.391].

### 2.2. Biochemical Analyses

Fifty-three percent of patients were at stages III and IV of the Fontaine classification, and 46% were at stages I and II. There were no significant differences between patients and controls with respect to age and smoking habit. PAD patients were more frequently male, and more suffered from arterial hypertension, diabetes mellitus, dyslipidemia, ischemic heart disease, and chronic obstructive pulmonary disease. There were significant increases in plasma galectin-3, F2-isoprostanes, CCL2, B2M, and CRP concentrations in PAD patients, compared with the control group (Table 1). Plasma galectin-3 and age were not significantly correlated (*r* = 0.142; *p* = 0.076). There were no significant differences in plasma galectin-3 concentrations when patients were segregated according to the other clinical and demographic variables (Table 2). We found significant direct correlations between plasma galectin-3 and F2-isoprostanes, CCL2, CRP, and B2M concentrations (Table 3).

**Figure 4:**
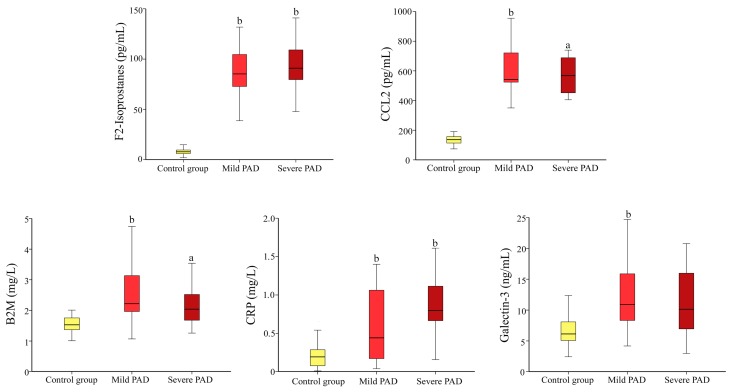
Selected biochemical variables in PAD patients classified according to whether they had mild disease (Fontaine Stages I and II) severe disease (Fontaine Stages III and IV), or were in the control group. Significance values by the Mann–Whitney *U* test: ^a^ *p* < 0.01; ^b^ *p* < 0.001, with respect to the control group.

We subsequently segregated PAD patients according to mild disease (Fontaine Stages I and II), or severe disease (Fontaine Stages III and IV); we did not observe any significant differences either in plasma galectin-3 concentrations, or in any of the other biochemical variables studied; the concentrations were increased with respect to the control group (Figure 4).

**Figure 5:**
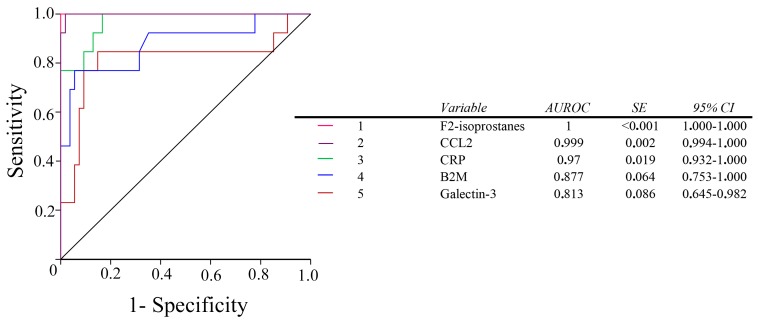
Receiver operating characteristics (ROC) plots for all the studied biomarkers in PAD patients and in the control group. AUROC: areas under the curve of the ROC plots. SE: Standard Error.

**Figure 6:**
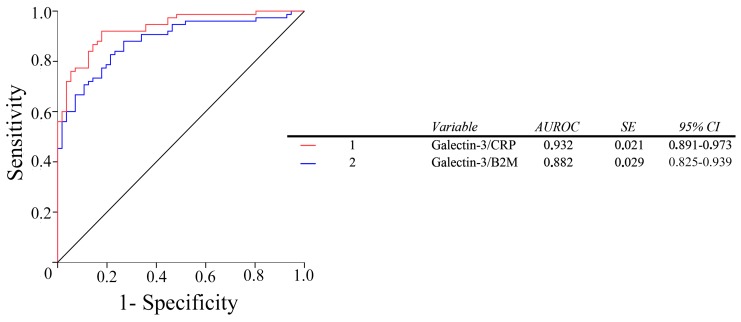
Receiver operating characteristics (ROC) plots for the galectin-3/CRP and galectin-3/B2M ratios in PAD patients and in the control group. AUROC: areas under the curve of the ROC plots. SE: Standard Error.

The diagnostic accuracy of all the selected variables in discriminating between the healthy volunteers, and the patients with PAD, was high. However, plasma galectin-3 measurement was not more efficient than the classical CRP and B2M markers. The order of the calculated accuracies was: F2-isoprostanes ≈ CCL2 > CRP ≈ B2M ≈ galectin-3 (Figure 5). The ratios between galectin-3 and B2M, or CRP significantly improved the diagnostic accuracy of galectin-3 alone (Figure 6).

## 3. Discussion

Galectins, particularly galectin-3, have been gaining importance recently as significant components within the cascade of events underlying the inflammatory reaction and fibrogenesis caused by oxidative stress [2]. Distributions in normal and diseased human peripheral arterial tissue have yet to be defined. Galectin-3 is known to be expressed in a variety of organs in the mouse including the kidney, lung, spleen, colon, uterus, and ovary [28]. In apolipoprotein E-deficient mice (a model of experimental atherosclerosis) galectin-3 is expressed in macrophage-rich areas, but not in smooth muscle cell-rich areas of aortic roots and brachiocephalic arteries [13]. Results of the present study show important differences in galectin-3 distribution in normal arteries, and arteries from PAD patients. Normal arteries showed galectin-3 expression exclusively in the adventitia, while arteries from patients showed the localization mainly adjacent to the smooth muscle cells of the media, with some minor staining in the adventitia and the intima. The pathophysiological interpretation of this finding cannot be inferred from the present investigation, but previous data suggest that this differential tissue distribution could be related to the processes of inflammation, fibrogenesis, and calcification of the atherosclerotic plaque. Indeed, patients with an advanced PAD and atheroma plaque expressed galectin-3 together with macrophages in the intima, suggesting a coordinated role in the formation of the plaque. This hypothesis is supported by data demonstrating that galectin-3 stimulates myofibroblast activation and fibrosis in several types of cells [29,30]. In aortic valves from patients with aortic stenosis, galectin-3 co-localized with the α-smooth muscle cell markers actin and vimentin. It also localized with osteogenic markers such as osteopontin, bone morphogenetic protein 2, runt-related transcription factor 2, and sex-determining region Y-box 9 [31]. Reports indicate that human carotid plaques express galectin-3 in smooth muscle cells, especially in macrocalcified areas, where it co-localizes with alkaline phosphatase [16]. Of note from an early study from our group is the observation of calcium deposition in the media of arteries from PAD patients; a distinct and frequent finding in affected arteries [32]. In the same study, we reported an increase in CD68-positive cells in diseased arteries; the conclusion drawn is that, under certain stimuli, arterial smooth muscle cells can shift their phenotypes to a macrophage-cell state [33]. In addition, another study from our group showed a similar distribution of CCL2 and its receptors in the intima of PAD patients with advanced disease and atheroma plaque [34]. In combination, these data strongly suggest that the changes in galectin-3 distribution in the arteries of patients with PAD are a component of the progression of, and molecular mechanisms underlying, the plaque formation. A caveat of the present study is that we cannot guarantee that the storage of the samples has not in any way altered the histological structure of the control, or diseased arteries. This possibility is unlikely, but it cannot be completely ruled out.

In contrast to the above, in performing quantitative analyses of galectin-3 expression, we found no significant differences between normal and PAD arteries. This could have resulted from the great difficulty in obtaining normal arteries. We used samples derived from tissue donors who were victims of a traffic accident. Selection for the present study followed histological analysis to exclude the presence of atherosclerosis. This resulted in lowering of the number of samples available and made the statistical analysis less than reliable. However, the confidence interval of galectin-3 staining measurements in patients with PAD covered the values observed in normal arteries. The postulation is that quantitative differences in expression between groups, if any, are not of high importance.

Alterations in plasma galectin-3 concentrations in PAD have been investigated by two independent research groups. Casanegra et al. [35] analyzed galectin-3 in 29 patients attending the Mayo Clinics with no evidence of PAD (normal ankle brachial index; ABI), and 31 patients with PAD (low ABI). They observed a mean galectin-3 concentration of 14.4 ng/mL in subjects with normal ABI, and approximately 22% increase in PAD patients. Our results are in the same range of measurement, albeit with some slight differences. Our values in the control group are lower, and the percentage increase in the patient group is approximately 76%. Demographic variation may account for these differences. In addition, the presence of concomitant diseases that could increase galectin-3 levels cannot be ruled-out in the control subjects of the Mayo Clinics’ study. Moreover, the PAD populations studied were different in that we had a higher frequency of patients with resting pain, ulceration, or gangrene. Madrigal-Matute et al. [7] reported plasma galectin-3 concentrations ranging between 2 and 20 ng/mL in PAD patients, with no significant difference with respect to the severity of the disease. This finding is concordant with our observation that plasma galectin-3 concentrations are similar in patients with moderate or severe PAD. They also observed that concentrations above the median were significantly associated with an increase in total mortality risk. A finding of note from our present study is the lack of association between plasma galectin-3 concentrations, and the presence of derangements such as arterial hypertension, diabetes, dyslipidemia, ischemic heart disease, or chronic obstructive pulmonary disease. A possible explanation could be that the interaction of PAD and galectin-3 is stronger than in these other diseases and, as such, masking the influence on the circulating levels of galectin-3. In addition, our patients had been receiving protracted treatment with numerous medications, which can also influence plasma galectin-3 concentrations in these concomitant diseases.

We observed direct correlations between plasma galectin-3 levels and markers of oxidative stress and inflammation, such as F2-isoprostanes, CCL2, CRP and B2M. Galectin-3 was shown [36] to induce oxidative stress through the release of O_2_^−^ in cultured mast cells, an effect that was blocked by the antioxidant enzyme superoxide dismutase. Galectin-3 is also expressed in human monocytes and released under NADPH oxidase-dependent superoxide synthesis [7]. In Wistar rats fed with a high-fat diet [37], leptin increased O_2_^−^ production by a mechanism that requires galectin-3. A recent prospective study in patients with chronic heart failure [38] showed direct correlations between galectin-3, markers of oxidative stress (oxidized low-density lipoproteins and extracellular superoxide dismutase), and markers of inflammation (CRP, interleukin-6), or heart failure (N-terminal pro b-type natriuretic peptide). Those findings are similar to these in the present investigation.

Several studies [18,35] have suggested that the measurement of plasma galectin-3 concentrations may be a good biomarker of diseases related to atherosclerosis. The results from the present investigation show that, in PAD, the diagnostic accuracy of this parameter, albeit quite high, is not superior to that of CRP and B2M, and lower to that of F2-isoprostanes and CCL2. The efficacy of F2-isoprostanes and CCL2 in discriminating between healthy individuals and PAD patients has already been reported by our group [27], and there are no other biochemical parameters identified to date that are superior in diagnostic accuracy. However, the ratios of galectin-3/CRP and galectin-3/B2M also showed a very high efficacy. Further, these measurements have the advantage over F2-isoprostanes, and CCL2, in that they can be easily automated and implemented in routine Clinical Chemistry laboratories, at a low cost and a speed of analysis [39,40,41]. Studies in wider series of patients need to be conducted to confirm the clinical usefulness of these ratios as PAD biomarkers.

## 4. Materials and Methods

### 4.1. Ethics Approval

The Hospital’s Ethics Committee (Institutional Review Board) approved the procedures of the study (approval documents 10-04-29/4proj3 and 2011-10-27/10proj1), and written informed consent was obtained from all participants.

### 4.2. Clinical Assessment of PAD Severity

The extent of PAD was determined using the Fontaine classification, which defines four stages: Stage I, asymptomatic; Stage II, intermittent claudication; Stage III, rest pain; Stage IV, ulceration or gangrene [42]. We also employed the ankle-brachial index (ABI), defined as the ratio of the systolic blood pressure at the ankle to that in the upper arm. Compared to the arm, lower blood pressure in the leg is an indication of blocked arteries due to PAD [43].

### 4.3. Participants

#### 4.3.1. Arteries from Normal Subjects and PAD Patients

We analyzed portions of femoral or popliteal arteries from patients obtained during surgical procedures for infra-inguinal limb revascularization in the Vascular Surgery Department of Hospital Universitari Joan XXIII between January 2014 and June 2016 (*n* = 50). All samples were from patients at Stages III and IV of the Fontaine classification. Four normal arteries obtained from accident victims between March 2014 and August 2015, and stored at the Blood and Tissue Bank of Catalonia (Banc de Sang i Teixits, www.bancsang.net/es/donants/donacio_teixits.html, Barcelona, Spain) were used as controls. All samples were stored at −80 °C until processed for histological examination.

#### 4.3.2. Characteristics of Subjects for the Biochemical Study

We underwent an observational, prospective, cross-sectional study in patients attending the Vascular Surgery Department of Hospital Universitari Joan XXIII. Diagnosis of PAD was performed by measuring the ABI, together with non-invasive imaging and angiography, when indicated. We recruited 86 patients with symptomatic PAD (79.1% men, 42–89 years old), 95% having an ABI between 0.4 and 0.9, and 5% with an ABI lower than 0.4. Degree of PAD was determined using the Fontaine classification; 2.6% of the patients were classified as Stage I; 43.6% as Stage II; 10.3% as Stage III; and 43.6% as Stage IV. Patients with clinical or analytical evidence of infection, acute ischemia, renal failure, liver disease, cancer, or autoimmune diseases were excluded. Diabetes, hypertension and dyslipidemia were defined according to established criteria.

The control group was composed of 72 plasma samples obtained from healthy volunteers participating in a population-based study (65.3% men, 58–79 years old). Participants in this study were randomly drawn from the local government census of three communities in the Mediterranean region of Tarragona (Northeast Spain). All members of the control group underwent a physical examination and a blood test. They could walk without any problems or pain and were ostensibly healthy with no clinical or analytical evidence of infectious disease, renal insufficiency, hepatic damage, neoplasia, oligophrenia, or dementia. Their serum concentrations of CRP and B2M were within the normal range. Medication intake was not an exclusion criterion, except in the case of drugs interfering with vitamin metabolism (methotrexate, tuberculostatics, theophylline, or vitamin B6 antagonists). The population studied did not consume vitamin supplements, or local food fortified with vitamins. Pregnant and recent post-partum women were not included in the study. All plasma and serum samples were collected between 2014 and 2016, and stored at −80 °C in our Biological Sample Bank.

### 4.4. Histological and Immunohistochemical Study

Arteries were rinsed in phosphate buffer to remove residual blood, and placed in at least 10 volumes of buffered formalin using a standard protocol for embedding tissue in paraffin wax for subsequent histology slide preparation. Three sections per slide were used for histological and immunohistochemical analyses. Sections, of 4-μm thickness, were stained with hematoxylin-eosin for histology. The intima and media thicknesses were measured in all histological sections as an estimate of the extent of atherosclerosis. The immunohistochemical expression of galectin-3 was analyzed using goat antibodies against human galectin-3 (dilution of 1/400) from R&D Systems, Inc. (Minneapolis, MN, USA). The appropriate biotinylated secondary antibodies, (Vector Laboratories Inc., Burlingame, CA, USA) were used at a dilution of 1:200. Detection was performed with the ABC peroxidase system (Vector Laboratories, Burlingame, CA, USA), and 3,3′-diaminobenzidine (DAB) peroxidase substrate (Dako Agilent Technologies, Glostrup, Denmark). All immunohistochemical sections were counterstained with Mayer’s hematoxylin. The positively-stained areas were quantified automatically (AnalySIS image software system), using an image analysis software (Soft Imaging System, Munster, Germany), and expressed as percentages of the total area. Control tissue samples were processed identically to the test samples, except that the primary antibodies were omitted from the incubation. The immunohistochemical expressions of CD68 antigen and α-actin were used as markers of macrophages and smooth muscle cells, respectively, and analyzed as previously reported [32,44].

### 4.5. Biochemical Assessments

Concentrations of galectin-3 were measured in the ethylene diamine tetraacetate (EDTA)-plasma using enzyme immunoassay (R&D Systems^®^, Minneapolis, MN, USA); Serum F2-isoprostanes and CCL2 were determined by enzyme immunoassay (Cayman Chemical Co., Ann Arbor, MI, USA, and Prepotech, London, UK, respectively). Serum high-sensitivity CRP and B2M concentrations were measured by automated immunoturbidimetry (Roche Diagnostics, Mannheim, Germany) in a Roche Modular Analytics P800 system (Roche Diagnostics, Basel, Switzerland).

### 4.6. Statistical Analyses

Differences between any two groups were assessed with the *χ*^2^ test (categorical) or the Mann–Whitney *U* test (continuous), since most of the variables studied had non-parametric distributions. The Spearman correlation coefficient was used to evaluate the degree of association between variables. The diagnostic accuracy of the measured biochemical variables was assessed by ROC curves. This analysis represents plots of all the sensitivity/specificity pairs resulting from varying decision thresholds. Sensitivity (or true positive rate) is the proportion of the sample correctly identified as being specific to the disease. Specificity (or true negative rate) is the proportion of subjects correctly identified as not being specific to the disease. The false positive rate was calculated as 1-specificity. The area under the curve (AUROC) and 95% confidence interval (CI) were calculated. The AUROC represents the ability of the test to correctly classify patients, with respect to the investigated parameter alteration. The values of AUROC can range between 1 (“perfect” test) and 0.5 (“worthless” test) [45].

## 5. Conclusions

We observed differences in the expression of galectin-3 in normal arteries, and arteries of patients with PAD, with a displacement of the expression from the adventitia to the media and the intima, which may suggest involvement of galectin-3 in the site of the atherosclerosis plaque formation. In addition, plasma galectin-3 concentration was increased in PAD patients, and correlated with serologic markers of oxidative stress and inflammation. The measurement of galectin-3 had a similar diagnostic accuracy to that of CRP and B2M, in the diagnosis of PAD. However, ratios of galectin-3/CRP and galectin-3/B2M showed improved usefulness as biomarkers in PAD.

## Figures and Tables

**Table 1:** Demographic, clinical, and biochemical characteristics of the control group and peripheral artery disease (PAD) patients.

Variable	Control Group (*n* = 72)	PAD (*n* = 86)	*p*-Value
Age, years	63 (59–73)	66 (49–87)	0.529
Male gender, *n* (%)	47 (65.3)	68 (79.1)	0.039
Smoking, *n* (%)	17 (23.6)	6 (10.9)	0.048
Medications, *n* (%)			
Antiplatelet drugs	Not recorded	25 (29.0)	
Statins		48 (55.8)	
Antidiabetic drugs		48 (55.8)	
Angiotensin converting enzyme inhibitors		48 (55.8)	
Angiotensin receptor antagonists		41 (47.6)	
Calcium receptor antagonists		55 (63.9)	
Diuretics		46 (53.5)	
Anti-arrhythmia drugs		73 (84.9)	
Beta-blockers		73 (84.9)	
Bronchodilators		59 (68.6)	
Arterial hypertension, *n* (%)	12 (16.9)	28 (63.6)	<0.001
Diabetes mellitus, *n* (%)	4 (5.6)	29 (67.4)	<0.001
Dyslipidemia, *n* (%)	7 (9.9)	20 (46.5)	<0.001
Ischemic heart disease, *n* (%)	0	4 (22.2)	
Chronic obstructive pulmonary disease, *n* (%)	0	9 (25.0)	
Ankle brachial index = 0.4–0.9	Not applicable	81 (94.2)	
Ankle brachial index < 0.4	Not applicable	5 (5.8)	
Fontaine classification			
Stage I, *n* (%)	Not applicable	3 (3.4)	
Stage II, *n* (%)	Not applicable	37 (43.0)	
Stage III, *n* (%)	Not applicable	9 (10.5)	
Stage IV, *n* (%)	Not applicable	37 (43.0)	
Galectin-3, ng/mL	6.13 (3.05–12.2)	10.79 (4.21–19.09)	<0.001
F2-isoprostanes, pg/mL	7.76 (3.03–14.82)	90.91 (47.62–141.71)	<0.001
Chemokine (C–C motif) ligand 2, pg/mL	136.34 (88.37–203.22)	565.75 (211.00–1154.00)	<0.001
β-2-microglobulin, mg/L	1.53 (1.09–2.35)	2.22 (1.34–4.55)	<0.001
C-reactive protein, mg/L	0.19 (0.02–0.74)	0.80 (0.07–2.82)	<0.001

**Table 2:** Plasma galectin-3 concentrations (ng/mL) in PAD patients segregated according to the presence or absence of the selected clinical variables.

Variable	No	Yes	*p*-Value
Male gender	11.22 (5.50–19.13)	10.60 (4.07–19.98)	0.361
Smoking	10.84 (4.57–19.00)	10.53 (18.89–26.55	0.948
Arterial hypertension	8.79 (3.29–16.76)	10.64 (5.13–23.12)	0.222
Diabetes mellitus	10.57 (3.29–24.69)	10.18 (4.08–19.00)	0.726
Dyslipidemia	10.10 (3.46–20.61)	10.20 (4.11–24.41)	0.932
Ischemic heart disease	8.79 (6.55–18.31)	9.43 (4.88–24.69)	0.878
Chronic obstructive pulmonary disease	9.72 (4.06–22.14)	11.45 (3.29–21.19)	0.349

**Table 3:** Correlations between plasma galectin-3 concentrations and the other selected biochemical variables.

Parameter	Spearman’s ρ	*p*-Value
F2-isoprostanes	0.437	<0.001
Chemokine (C−C motif) ligand	0.295	0.005
C-reactive protein	0.341	<0.001
β-2-microglobulin	0.544	<0.001

## References

[1] Barondes S.H., Cooper D.N., Gitt M.A., Leffler H. (1994). Galectins. Structure and function of a large family of animal lectins. J. Biol. Chem..

[2] Pugliese G., Iacobini C., Pesce C.M., Menini S. (2015). Galectin-3: An emerging all-out player in metabolic disorders and their complications. Glycobiology.

[3] Rubinstein N., Ilarregui J.M., Toscano M.A., Rabinovich G.A. (2004). The role of galectins in the initiation, amplification and resolution of the inflammatory response. Tissue Antigens.

[4] Paz I., Sachse M., Dupont N., Mounier J., Cederfur C., Enninga J., Leffler H., Poirier F., Prevost M.C., Lafont F. (2010). Galectin-3, a marker for vacuole lysis by invasive pathogens. Cell. Microbiol..

[5] DeRoo E.P., Wrobleski S.K., Shea E.M., Al-Khalil R.K., Hawley A.E., Henke P.K., Myers D.D., Wakefield T.W., Diaz J.A. (2015). The role of galectin-3 and galectin-3-binding protein in venous thrombosis. Blood.

[6] Diaz J.A., Ramacciotti E., Wakefield T.W. (2010). Do galectins play a role in venous thrombosis? A review. Thromb. Res..

[7] Madrigal-Matute J., Lindholt J.S., Fernandez-Garcia C.E., Benito-Martin A., Burillo E., Zalba G., Beloqui O., Llamas-Granda P., Ortiz A., Egido J. (2014). Galectin-3, a biomarker linking oxidative stress and inflammation with the clinical outcomes of patients with atherothrombosis. J. Am. Heart Assoc..

[8] Chen S.C., Kuo P.L. (2016). The role of galectin-3 in the kidneys. Int. J. Mol. Sci..

[9] Wang L., Guo X.L. (2016). Molecular regulation of galectin-3 expression and therapeutic implication in cancer progression. Biomed. Pharmacother..

[10] Menini S., Iacobini C., Blasetti Fantauzzi C., Pesce C.M., Pugliese G. (2016). Role of galectin-3 in obesity and impaired glucose homeostasis. Oxid. Med. Cell Longev..

[11] Lala R.I., Puschita M., Darabantiu D., Pilat L. (2015). Galectin-3 in heart failure pathology—“Another brick in the wall”?. Acta Cardiol..

[12] Nachtigal M., Ghaffar A., Mayer E.P. (2008). Galectin-3 gene inactivation reduces atherosclerotic lesions and adventitial inflammation in ApoE-deficient mice. Am. J. Pathol..

[13] Papaspyridonos M., McNeill E., de Bono J.P., Smith A., Burnand K.G., Channon K.M., Greaves D.R. (2008). Galectin-3 is an amplifier of inflammation in atherosclerotic plaque progression through macrophage activation and monocyte chemoattraction. Arterioscler. Thromb. Vasc. Biol..

[14] MacKinnon A.C., Liu X., Hadoke P.W., Miller M.R., Newby D.E., Sethi T. (2013). Inhibition of galectin-3 reduces atherosclerosis in apolipoprotein E-deficient mice. Glycobiology.

[15] Yu L., Ruifrok W.P., Meissner M., Bos E.M., van Goor H., Sanjabi B., van der Harst P., Pitt B., Goldstein I.J., Koerts J.A. (2013). Genetic and pharmacological inhibition of galectin-3 prevents cardiac remodeling by interfering with myocardial fibrogenesis. Circ. Heart Fail..

[16] Menini S., Iacobini C., Ricci C., Blasetti F.C., Salvi L., Pesce C.M., Relucenti M., Familiari G., Taurino M., Pugliese G. (2013). The galectin-3/RAGE dyad modulates vascular osteogenesis in atherosclerosis. Cardiovasc. Res..

[17] Sharma U.C., Pokharel S., van Brakel T.J., van Berlo J.H., Cleutjens J.P., Schroen B., Andre S., Crijns H.J., Gabius H.J., Maessen J. (2004). Galectin-3 marks activated macrophages in failure-prone hypertrophied hearts and contributes to cardiac dysfunction. Circulation.

[18] Salvagno G.L., Pavan C. (2016). Prognostic biomarkers in acute coronary syndrome. Ann. Trans. Med..

[19] Hashmi S., Al-Salam S. (2015). Galectin-3 is expressed in the myocardium very early post-myocardial infarction. Cardiovasc. Pathol..

[20] Sharma U.C., Mosleh W., Chaudhari M.R., Katkar R., Weil B., Evelo C., Cimato T.R., Pokharel S., Blankesteijn W.M., Suzuki G. (2016). Myocardial and serum galectin-3 expression dynamics marks post-myocardial infarction cardiac remodelling. Heart Lung Circ..

[21] Criqui M.H. (2001). Peripheral arterial disease–epidemiological aspects. Vasc. Med..

[22] Strzyżewski K.W., Pioruńska-Stolzmann M., Majewski W., Kasprzak M., Strzyżewski W. (2013). Effect of surgical treatment on lipid peroxidation parameters and antioxidant status in the serum of patients with peripheral arterial disease. Dis. Markers.

[23] Rull A., Camps J., Alonso-Villaverde C., Joven J. (2010). Insulin resistance, inflammation, and obesity: Role of monocyte chemoattractant protein-1 (or CCL2) in the regulation of metabolism. Mediat. Inflamm..

[24] Rull A., García R., Fernández-Sender L., Beltrán-Debón R., Aragonès G., Alegret J.M., Alonso-Villaverde C., Mackness B., Mackness M., Camps J. (2011). The role of combined assessment of defense against oxidative stress and inflammation in the evaluation of peripheral arterial disease. Curr. Mol. Med..

[25] McDermott M.M., Lloyd-Jones D. (2009). The role of biomarkers and genetics in peripheral artery disease. J. Am. Coll. Cardiol..

[26] Cooke J.P., Wilson A.M. (2010). Biomarkers of peripheral artery disease. J. Am. Coll. Cardiol..

[27] Fort-Gallifa I., García-Heredia A., Hernández-Aguilera A., Simó J.M., Sepúlveda J., Martín-Paredero V., Camps J., Joven J. (2016). Biochemical indices of oxidative stress and inflammation in the evaluation of peripheral artery disease. Free Radic. Biol. Med..

[28] Kim H., Lee J., Hyun J.W., Park J.W., Joo H.G., Shin T. (2007). Expression and immunohistochemical localization of galectin-3 in various mouse tissues. Cell. Biol. Int..

[29] Henderson N.C., Mackinnon A.C., Farnworth S.L., Poirier F., Russo F.P., Iredale J.P., Haslett C., Simpson K.J., Sethi T. (2006). Galectin-3 regulates myofibroblast activation and hepatic fibrosis. Proc. Natl. Acad. Sci. USA.

[30] Qian X., Li M., Wagner M.B., Chen G., Song X. (2016). Doxazosin stimulates galectin-3 expression and collagen synthesis in HL-1 cardiomyocytes independent of protein kinase C pathway. Front. Pharmacol..

[31] Sádaba J.R., Martínez-Martínez E., Arrieta V., Álvarez V., Fernández-Celis A., Ibarrola J., Melero A., Rossignol P., Cachofeiro V., López-Andrés N. (2016). Role for galectin-3 in calcific aortic valve stenosis. J. Am. Heart Assoc..

[32] Rull A., Hernandez-Aguilera A., Fibla M., Sepulveda J., Rodríguez-Gallego E., Riera-Borrull M., Sirvent J.J., Martín-Paredero V., Menendez J.A., Camps J. (2014). Understanding the role of circulating chemokine (C–C motif) ligand 2 in patients with chronic ischemia threatening the lower extremities. Vasc. Med..

[33] Rong J.X., Shapiro M., Trogan E., Fisher E.A. (2003). Transdifferentiation of mouse aortic smooth muscle cells to a macrophage-like state after cholesterol loading. Proc. Natl. Acad. Sci. USA.

[34] Hernández-Aguilera A., Sepúlveda J., Rodríguez-Gallego E., Guirro M., García-Heredia A., Cabré N., Luciano-Mateo F., Fort-Gallifa I., Martín-Paredero V., Joven J. (2015). Immunohistochemical analysis of paraoxonases and chemokines in arteries of patients with peripheral artery disease. Int. J. Mol. Sci..

[35] Casanegra A.I., Stoner J.A., Tafur A.J., Pereira H.A., Rathbun S.W., Gardner A.W. (2016). Differences in galectin-3, a biomarker of fibrosis, between participants with peripheral artery disease and participants with normal ankle-brachial index. Vasc. Med..

[36] Suzuki Y., Inoue T., Yoshimaru T., Ra C. (2008). Galectin-3 but not galectin-1 induces mast cell death by oxidative stress and mitochondrial permeability transition. Biochim. Biophys. Acta.

[37] Martínez-Martínez E., Jurado-López R., Valero-Muñoz M., Bartolomé M.V., Ballesteros S., Luaces M., Briones A.M., López-Andrés N., Miana M., Cachofeiro V. (2014). Leptin induces cardiac fibrosis through galectin-3, mTOR and oxidative stress: Potential role in obesity. J. Hypertens..

[38] Medvedeva E.A., Berezin I.I., Surkova E.A., Yaranov D.M., Shchukin Y.V. (2016). Galectin-3 in patients with chronic heart failure: Association with oxidative stress, inflammation, renal dysfunction and prognosis. Minerva Cardioangiol..

[39] La’ulu S.L., Apple F.S., Murakami M.M., Ler R., Roberts W.L., Straseski J.A. (2013). Performance characteristics of the ARCHITECT Galectin-3 assay. Clin. Biochem..

[40] Gruson D., Mancini M., Ahn S.A., Rousseau M.F. (2014). Measurement of Galectin-3 with the ARCHITECT assay: Clinical validity and cost-effectiveness in patients with heart failure. Clin. Biochem..

[41] Gruson D., Mancini M., Ahn S.A., Rousseau M.F. (2014). Galectin-3 testing: Validity of a novel automated assay in heart failure patients with reduced ejection fraction. Clin. Chim. Acta.

[42] Becker F. (1985). Exploration of arterial function with noninvasive technics. Results in chronic arterial occlusive disease of the lower limbs according to Leriche and Fontaine classification. Int. Angiol..

[43] Al-Qaisi M., Nott D.M., King D.H., Kaddoura S. (2009). Ankle brachial pressure index (ABPI): An update for practitioners. Vasc. Health Risk Manag..

[44] Krishnan P., Purushothaman K.R., Purushothaman M., Turnbull I.C., Tarricone A., Vasquez M., Jain S., Baber U., Lascano R.A., Kini A.S. (2016). Enhanced neointimal fibroblast, myofibroblast content and altered extracellular matrix composition: Implications in the progression of human peripheral artery restenosis. Atherosclerosis.

[45] Zweig M.H., Campbell G. (1993). Receiver-operating characteristics (ROC) plots: A fundamental evaluation tool in clinical medicine. Clin. Chem..

